# Mechanical Performance Investigation of Recycled HDPE Reinforced with Nanoclay for Enhanced Strength and Sustainability

**DOI:** 10.3390/polym18131615

**Published:** 2026-06-29

**Authors:** Sundarakannan Rajendran, Sakthivel Sankaran, Geetha Palani, Magdalena Niemczewska-Wójcik, Thirumalai Kumaran Sundaresan, Uthayakumar Marimuthu, Koppiahraj Karuppiah

**Affiliations:** 1 Department of Mechanical Engineering, Vel Tech Rangarajan Dr.Sagunthala R&D Institute of Science and Technology, Avadi, Chennai 600062, Tamil Nadu, India; drsundarakannanr@veltech.edu.in; 2 Department of Biomedical Engineering, Kalasalingam Academy of Research and Education, Krishnankovil 626126, Tamil Nadu, India; sakthivel@klu.ac.in; 3 Department of Physics, Rajalakshmi Engineering College, Thandalam, Sriperumbudur, Chennai 602105, Tamil Nadu, India; geethapalani@rajalakshmi.edu.in; 4 Faculty of Mechanical Engineering, Cracow University of Technology, Jana Pawła II No. 37, 31-864 Krakow, Poland; magdalena.niemczewska-wojcik@pk.edu.pl; 5 Department of Mechanical Engineering, PSG Institute of Technology and Applied Research, Coimbatore 641062, Tamil Nadu, India; thirumalaikumaran@psgitech.ac.in; 6 Department of Mechanical Engineering, Center for Flexible Electronics & Advanced Materials, Amrita Vishwa Vidyapeetham, Amritapuri, Kollam 690525, Kerala, India; uthay@am.amrita.edu; 7 Department of Mechanical Engineering, KIT-Kalaignarkarunanidhi Institute of Technology, Coimbatore 641402, Tamil Nadu, India

**Keywords:** recycled plastics, nanoparticles, sustainability, mechanical properties, nanocomposites

## Abstract

The increasing demand for sustainable materials has intensified efforts to enhance the performance of recycled polymers for engineering applications. This study investigates the effect of nanoclay reinforcement on the mechanical properties of recycled high-density polyethylene (rHDPE). Nanoclay was incorporated into rHDPE at varying loadings through melt blending, and the resulting composites were evaluated in terms of tensile, flexural, impact, and hardness properties. The tensile strength and tensile modulus improved significantly with increasing nanoclay content, reaching maximum values of 31.27 MPa and 2.39 GPa, respectively, at 1.5 wt% nanoclay, corresponding to increases of 23.11% and 47.53% relative to unreinforced rHDPE. Similarly, the flexural strength and flexural modulus attained peak values of 25.88 MPa and 1105.08 MPa at 1.5 wt% nanoclay, representing improvements of 12.57% and 15.49%, respectively. Impact strength exhibited a different trend, achieving a maximum value of 73.58 kJ/m^2^ at 0.5 wt% nanoclay before decreasing at higher loadings, indicating a transition towards more brittle behaviour. Hardness increased progressively with nanoclay addition and reached a maximum value of 68.06 Shore D at 1.5 wt%, exceeding both unreinforced rHDPE and virgin HDPE. The overall results demonstrate that nanoclay effectively compensates for the mechanical degradation associated with recycling by enhancing stiffness, strength, and surface hardness. Among the investigated formulations, 1.5 wt% nanoclay provided the optimum balance of mechanical performance, while higher loadings led to reduced reinforcement efficiency due to particle agglomeration. These findings highlight the potential of nanoclay-reinforced rHDPE as a sustainable, high-performance material for applications in packaging, construction, and automotive components, thereby supporting circular economy initiatives and resource-efficient material development.

## 1. Introduction

Plastic waste has become a pressing global issue due to its detrimental environmental impact and increasing accumulation in landfills and oceans. Plastic waste is generated globally at a rate of approximately 400 million metric tonnes per year [1], with High-Density Polyethylene (HDPE) playing a significant role due to its widespread use in packaging. Packaging materials account for 46% of total plastic waste, indicating that HDPE is the dominant material in this sector [2]. Despite its prevalence, only about 9% of all plastic waste is recycled globally, with the recycling rate for HDPE natural bottles in the United States reaching 29.3% in 2018 [3]. In India, plastic packaging recycling targets for FY 2024/25 have been set between 30% and 50%, depending on the product category, reflecting efforts to increase recycling [4]. HDPE is non-biodegradable and can take 450 to 1000 years to decompose, resulting in long-term environmental persistence. In this context, mechanical recycling of HDPE represents a critical strategy for reducing plastic waste and conserving petrochemical resources, and its importance has been further reinforced by growing regulatory and market pressure toward circular economy objectives [5]. A recent comprehensive review by Rasilainen et al. [6] confirmed that nanocomposite fabrication is among the most effective strategies for transforming recycled plastic waste into value-added materials, as nanomaterial reinforcement can recover and, in some cases, surpass the mechanical, thermal, and barrier properties lost during conventional mechanical recycling.

However, recycled HDPE (rHDPE) often exhibits inferior mechanical, thermal, and barrier properties compared to virgin HDPE, largely due to thermal and oxidative degradation during the recycling process [7,8,9]. The dominant degradation mechanisms, chain scission and thermo-oxidative branching, reduce the molecular weight of the polymer and disrupt its crystalline microstructure, resulting in measurable losses in tensile strength, stiffness, and impact resistance [10]. These property penalties progressively compound with successive reprocessing cycles, limiting the substitutability of rHDPE in demanding engineering and industrial sectors. Blending recycled HDPE with virgin material can partially compensate for these losses [11], but blending alone is insufficient to recover the full mechanical performance required for high-value applications such as automotive components, structural construction profiles, and rigid industrial packaging.

To overcome these limitations, reinforcing recycled HDPE with nanoparticles has emerged as a promising and scalable strategy. Nanoparticles such as silica, graphene, carbon nanotubes (CNTs), nanoclay, and aluminium oxide possess unique properties, including high aspect ratios, superior mechanical strength, and thermal conductivity, that can significantly improve the properties of polymer matrices [12,13,14]. These nanomaterials enhance tensile strength, impact resistance, thermal stability, and barrier properties of recycled HDPE through mechanisms such as load transfer, crack deflection, and thermal barrier effects [15,16]. Additionally, the high surface area of nanoparticles allows for better interfacial adhesion with the polymer matrix, further contributing to property enhancement [17,18]. Among available reinforcing options, silica nanoparticles have been shown to increase the tensile strength of HDPE matrices at low loadings of 0.75 vol%, with further additions causing agglomeration-induced reductions, confirming the importance of loading optimisation [19]. Similarly, graphene additions of 0.5–1 wt% to recycled HDPE and high-molecular-weight polyethylene blends improved Young’s modulus and tensile strength without compromising stress-crack resistance, demonstrating the applicability of nano-reinforcement in industrially relevant recycled polyethylene grades [20]. Nano-alumina reinforcement of recycled HDPE has likewise been explored for packaging applications, with scanning electron microscopy confirming effective filler distribution at optimal concentrations [21]. Among available nanofillers, organically modified montmorillonite (nanoclay) is particularly attractive due to its natural abundance, low cost, established surface modification chemistry, and proven compatibility with polyolefin matrices when maleic-anhydride-grafted polyethylene compatibilisers are used to promote clay exfoliation and filler–matrix interfacial bonding [22].

The effectiveness of nanofillers in improving recycled polymer properties has been reported by several researchers. Chen et al. [23] investigated the effects of organoclay on the mechanical performance of recycled HDPE/PET composites and reported that low nanofiller additions can improve stiffness and strength, whereas excessive loading may promote agglomeration and reduce performance. These observations support the need to optimise filler loading when designing recycled polymer nanocomposites for structural applications. Sánchez-Valdés et al. [22] demonstrated that blends of virgin and recycled HDPE reinforced with nanoclay exhibited significant improvements in tensile, flexural, and impact properties when an amine-alcohol-modified HDPE compatibiliser was used, outperforming conventional maleic-anhydride-grafted systems and highlighting the critical role of interfacial engineering. In a related study investigating recycled polyethylene composites, Sadik et al. [24] showed that the combined use of nanosilica and nanoclay in recycled polyethylene/rice husk composites produced the greatest mechanical improvement on nanoclay loading, with higher concentrations leading to clay agglomeration that blocked efficient stress transfer, a finding directly relevant to the optimisation approach taken in the present work. Furthermore, Sahu et al. [25] reported that rHDPE reinforced with 4 wt% carbon nanotubes exhibited a 39% increase in tensile strength compared to pure rHDPE, while Malyuta et al. [26] reported that talc-reinforced rHDPE achieved up to 20.4% improvement in tensile strength alongside substantial gains in elastic stiffness, collectively supporting the premise that low-loading nanofiller reinforcement is a broadly effective route to recovering and enhancing the mechanical performance of recycled polyolefins.

In this study, recycled HDPE was reinforced with varying concentrations of nanoclay at loadings of 0.5, 1.0, 1.5, and 2.0 wt% using the melt blending method. The nanocomposites were characterised using a universal testing machine for mechanical properties. The study aims to systematically evaluate the influence of nanoparticle loading and dispersion quality on composite properties and identify optimal formulations for various industrial applications. A cradle-to-gate life cycle assessment was additionally conducted to quantify the environmental burdens of each formulation and identify the most sustainable composite under a multi-criteria framework. The findings of this research are expected to provide a robust framework for transforming recycled HDPE into high-performance polymer composites, enabling its use in automotive parts, structural components, and packaging materials. By demonstrating the feasibility of using nanotechnology to enhance recycled polymers, this work contributes to the advancement of sustainable material development and supports the principles of the circular economy.

## 2. Materials and Methods

### 2.1. Materials

Recycled high-density polyethylene (rHDPE) was sourced from post-consumer waste (water cap bottles), cleaned, and pelletized. The recycled HDPE possessed a melt flow index (MFI) of approximately 0.75 g/10 min (190 °C/2.16 kg). Montmorillonite bentonite nanoclay (Nanoshel, Intelligent Materials Pvt Ltd, Punjab, India; purity 99.9%) was used as the reinforcing nanofiller. The nanoclay possessed an average particle thickness of ca. 1 nm with high aspect-ratio layered silicate morphology suitable for polymer reinforcement applications. According to the supplier specification sheet, the material had a specific gravity of 2.6, surface area of 0.09–1.8 m^2^/cc, and pH range of 8.5–10.5. The nanoscale layered structure and high surface area of montmorillonite contributed to improved filler–matrix interaction and reinforcement efficiency in the rHDPE matrix. Maleic anhydride-grafted polyethylene (MAH-g-PE) was used as a compatibilizer at 1 wt% to improve interfacial adhesion between the hydrophilic nanoclay and hydrophobic rHDPE matrix.

### 2.2. Composite Fabrication

The fabrication of rHDPE composites involved melt compounding followed by injection moulding. During melt compounding, rHDPE pellets and nanoclay particles at varying weight percentages (0.5 wt%, 1 wt%, 1.5 wt%, and 2 wt%) were pre-dried at 60 °C for 5 h to eliminate moisture and prevent processing defects. MAH-g-PE compatibilizer was incorporated to enhance compatibility between the nanoclay and polymer matrix. A twin-screw extruder was used for compounding due to its superior mixing capability. The barrel temperature was maintained between 190 °C and 210 °C to ensure complete melting of rHDPE without thermal degradation of the polymer or nanoparticles. The extrusion process was carried out at a screw speed of 60 rpm with an average residence time of approximately 4–5 min to achieve homogeneous nanoclay dispersion within the matrix. The compounded material was extruded into strands, cooled in a water bath, and pelletized into composite granules.

**Figure 1:**
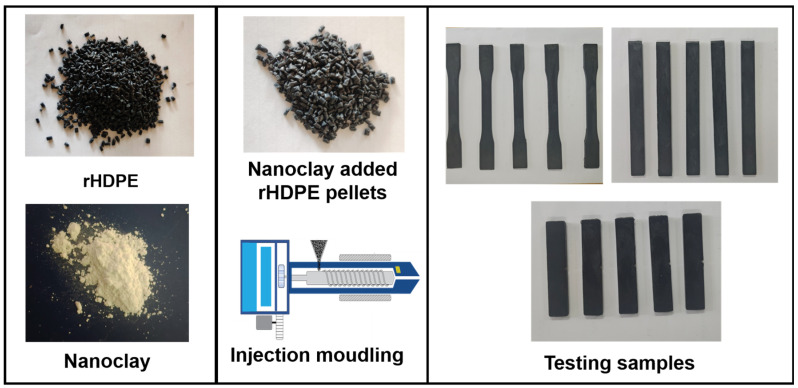
Materials and testing samples fabricated.

The granules were subsequently processed using injection moulding to fabricate test specimens. Prior to moulding, the granules were re-dried at 60 °C and fed into the hopper of the injection moulding machine. The barrel temperature ranged from 200 °C to 220 °C, while the nozzle temperature was maintained at approximately 200 °C to ensure proper melt flow. The molten composite was injected into preheated moulds maintained at 50 °C under an injection pressure of 80 MPa, followed by a holding pressure of 45 MPa for 12 s to improve dimensional stability and reduce internal void formation. The moulded samples were cooled for approximately 35–40 s before ejection to minimize residual stresses and ensure dimensional consistency. The moulded specimens were then trimmed and polished according to ASTM standards for mechanical testing. These fabrication conditions ensured consistent specimen quality and effective reinforcement dispersion within the rHDPE matrix. Figure 1 shows the rHDPE granules and fabricated composite samples.

### 2.3. Testing

Tensile properties of the composites were evaluated in accordance with ASTM D638 using a Dak System Inc. Universal Testing Machine (U.T.M., MAEON Laboratories, Chennai, India) equipped with a 10 kN axial load cell. The specimens were tested with a gauge length of 50 mm and parallel length of 150 mm, and tensile strength, elongation at break, stress at break, peak load, and modulus were recorded. The specimens were prepared in a dog-bone shape with dimensions of 250 × 25 × 3 mm, and testing was conducted at a crosshead speed of 5 mm/min. Flexural properties were measured using a three-point bending configuration in accordance with ASTM D790. Specimens with dimensions of 127 × 12.7 × 3 mm were tested using a span-to-thickness ratio of 32:1 and a crosshead speed of 1 mm/min. Impact strength was assessed using a Charpy impact tester following ASTM D6110. Notched specimens measuring 64 × 12.7 × 3 mm were used to evaluate energy absorption under dynamic loading. Shore D hardness was measured according to ASTM D2240. These standardised methods ensured reliable and reproducible measurements of the mechanical properties under tensile, flexural, impact, and surface indentation loading conditions.

### 2.4. Life Cycle Assessment (LCA) Framework

A combined experimental–computational framework was employed to evaluate the mechanical, environmental, and sustainability performance of virgin HDPE, recycled HDPE (rHDPE), and rHDPE–nanoclay composites with filler loadings of 0.5–2.0 wt.%. Mechanical properties including tensile strength, flexural strength, impact energy, and Shore D hardness were compiled from experimental measurements and processed using a custom Python 3.12 script executed in Spyder [27]. The script performed normalization, sustainability index calculations, life-cycle impact modelling, uncertainty analysis, and graphical visualization of performance trends.

#### 2.4.1. System Boundary and Scope

A cradle-to-gate life cycle assessment was performed in accordance with ISO 14040/14044 standards to quantify the environmental impacts associated with each composite formulation. The system boundary encompassed four major processes: (i) raw material extraction, including virgin HDPE polymerisation and mining/purification of montmorillonite clay; (ii) material production and processing, incorporating HDPE reprocessing and nanoclay organo-modification stages; (iii) transport of raw materials to the compounding facility; and (iv) composite processing through melt blending and injection moulding. Excluded stages comprised the use phase, end-of-life treatment, disposal, and downstream distribution, as these are beyond the scope of material production assessment. The functional unit was defined as 1 kg of composite material ready for injection moulding, ensuring comparability across formulations.

The geographic scope represented global average data for virgin HDPE production and regionally representative values for recycled HDPE sourced from developing economies with established collection and reprocessing systems. Data quality followed ISO cutoff criteria, limiting any excluded mass flows to <1%. By isolating material production steps, the cradle-to-gate boundary allowed direct comparison of the environmental benefits associated with recycled content and the influence of low-percentage nanoclay reinforcement.

#### 2.4.2. Life Cycle Inventory (LCI) Data and Sources

Environmental inventory data for virgin HDPE, recycled HDPE, and nanoclay were sourced from Ecoinvent 3.9 and peer-reviewed LCA studies. Global warming potential (GWP, kg CO_2_-eq/kg) and primary energy demand (PED, MJ/kg) were selected as the core environmental indicators. Virgin HDPE production contributed GWP = 1.95 kg CO_2_-eq/kg (1.80–2.10) and PED = 82 MJ/kg (78–86). Recycled HDPE exhibited significantly lower burdens due to avoided petroleum extraction and polymerisation, with GWP = 0.48 kg CO_2_-eq/kg (0.43–0.53) and PED = 28.5 MJ/kg (25.7–31.3). Nanoclay production, incorporating ore extraction, purification, drying, and organo-modification, showed GWP = 1.15 kg CO_2_-eq/kg (1.04–1.26) and PED = 58 MJ/kg (52.2–63.8). Despite nanoclay’s relatively high per-unit environmental burden, its overall contribution to composite impact remained small due to the low filler loading (≤2 wt.%). A summary of the LCI values is presented in Table 1.

Composite processing energy associated with melt blending and injection moulding was represented by a simplified processing-energy factor equivalent to 6% of the material primary energy demand (PED), with a sensitivity range of 3–9%. This assumption was informed by the study of Hesser et al. [33], in which specific energy consumption values of approximately 1.6–3.5 MJ kg^−1^ were reported for the injection moulding of polypropylene-based composites. When expressed relative to the PED of rHDPE (28.5 MJ kg^−1^), these values corresponded to approximately 5.6–12.3% of the material energy content. Furthermore, injection moulding was reported to contribute only 4–7% of the cradle-to-gate non-renewable energy use of polypropylene and polypropylene-based composites, indicating that processing energy constitutes a relatively minor proportion of the overall environmental burden. Accordingly, a central value of 6% was adopted as a conservative estimate representative of an energy-efficient processing scenario for the small-batch compounding and injection moulding of polyolefin nanocomposites. To evaluate the robustness of this assumption, a sensitivity analysis covering a broader range of 3–9% was performed. It was thereby confirmed that variations in processing-energy allocation within this range did not alter the relative sustainability ranking of the investigated formulations. This simplifying assumption was informed by published specific energy consumption ranges for injection moulding and extrusion-related polymer processing, which show substantial variability depending on machine scale, throughput, and operating conditions [33]. Processing-related GWP was calculated separately from the electricity demand of 0.12 kWh/kg and the India-grid carbon intensity of 0.82 kg CO_2_-eq/kWh [34], yielding an additive processing GWP contribution of 0.0984 kg CO_2_-eq/kg. The total GWP of each formulation was therefore computed as the weighted material GWP plus the additive processing GWP, while PED was computed as the weighted material PED multiplied by the 1.06 processing-energy factor.

(1) $$\begin{array}{cccc} & \mathrm{Total}\;\mathrm{GWP}=\left(w_{m}\cdot {\mathrm{GWP}}_{m}+w_{c}\cdot {\mathrm{GWP}}_{c}\right)+0.0984 & & \end{array}$$

(2) $$\begin{array}{cccc} & \mathrm{Total}\;\mathrm{PED}=\left(w_{m}\cdot {\mathrm{PED}}_{m}+w_{c}\cdot {\mathrm{PED}}_{c}\right)\times 1.06 & & \end{array}$$

where $w_{m}$ and $w_{c}$ are the mass fractions of the matrix and nanoclay, respectively; ${\mathrm{GWP}}_{m}$ and ${\mathrm{PED}}_{m}$ are the global warming potential and primary energy demand of the matrix; and ${\mathrm{GWP}}_{c}$ and ${\mathrm{PED}}_{c}$ are the corresponding values for nanoclay.

The two primary environmental indicators used in this study were global warming potential, expressed in kg ${Co}_{2}$-eq/kg composite, and primary energy demand, expressed in MJ/kg composite. These values were normalised to enable integration with mechanical performance metrics.

#### 2.4.3. Multi-Criteria Sustainability Assessment Framework

To unify environmental and mechanical performance, a Sustainability Index (SI) was developed comprising three sub-indices. Each mechanical property was normalised between 0 and 1 using min-max scaling:

(3) $$\begin{array}{cccc} & P_{\mathrm{norm}}=\frac{P_{\mathrm{measured}}-P_{min}}{P_{max}-P_{min}} & & \end{array}$$

where $P_{\mathrm{measured}}$ is the experimentally measured property value, and $P_{min}$ and $P_{max}$ are the minimum and maximum values of that property within the dataset.

The Mechanical Performance Index was then calculated as the arithmetic mean of the four normalised mechanical properties:

(4) $$\begin{array}{cccc} & \mathrm{MechIndex}=\frac{T_{\mathrm{norm}}+F_{\mathrm{norm}}+I_{\mathrm{norm}}+H_{\mathrm{norm}}}{4} & & \end{array}$$

where $T_{\mathrm{norm}}$, $F_{\mathrm{norm}}$, $I_{\mathrm{norm}}$, and $H_{\mathrm{norm}}$ represent the normalised tensile strength, flexural strength, impact strength, and hardness, respectively.

The environmental indicators were similarly normalised and averaged to obtain the Environmental Burden Index:

(5) $$\begin{array}{cccc} & \mathrm{EnvIndex}=\frac{{\mathrm{GWP}}_{\mathrm{norm}}+{\mathrm{PED}}_{\mathrm{norm}}}{2} & & \end{array}$$

The Green Index was defined as:

(6) $$\begin{array}{cccc} & \mathrm{GreenIndex}=1-\mathrm{EnvIndex} & & \end{array}$$

Finally, the Sustainability Index was expressed as:

(7) $$\begin{array}{cccc} & \mathrm{SI}=0.5\times \mathrm{MechIndex}+0.5\times \mathrm{GreenIndex} & & \end{array}$$

Equal weighting (0.5:0.5) was selected to provide balanced consideration between material functionality and environmental sustainability, as both criteria are equally important for sustainable polymer composite applications. In addition, weighting sensitivity analysis was performed to evaluate the robustness of the Sustainability Index under varying mechanical–environmental priority conditions. Materials with higher SI values represent more favourable trade-offs between mechanical performance and environmental impact.

#### 2.4.4. Uncertainty and Sensitivity Analysis

Uncertainty in LCA inputs was represented using source-based ranges of ±7.7% for virgin HDPE, ±10% for recycled HDPE and nanoclay, and ±6% for processing electricity. In the composite-level uncertainty bounds, the lower and upper GWP limits were estimated by combining the material-specific uncertainty ranges with the fixed processing contribution. A broader scenario-based sensitivity analysis was then performed by varying the environmental inputs by ±20% and by changing the relative weighting between mechanical and environmental performance.

All calculations were performed using Python 3.12 in Spyder IDE [27]. Data handling, numerical analysis, and visualisation were carried out using pandas, NumPy, and Matplotlib, respectively. The software versions used were Python 3.12, Spyder 6.0, pandas 2.2, NumPy 2.0, and Matplotlib 3.9.

## 3. Results and Discussion

### 3.1. Tensile Strength

**Figure 2:**
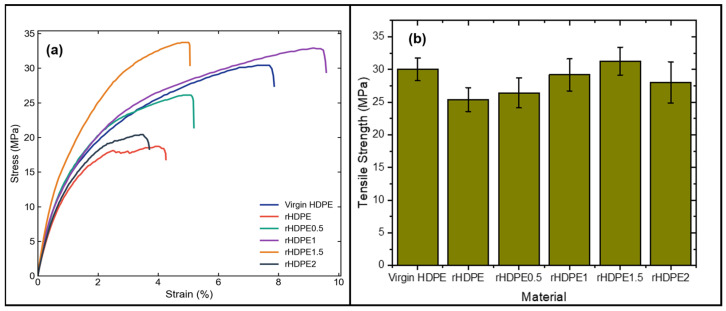
(**a**) Stress–strain graph and (**b**) Tensile strength of rHDPE composites.

Figure 2a presents the stress–strain behaviour of virgin HDPE, recycled HDPE (rHDPE), and rHDPE reinforced with varying nanoclay loadings, while the corresponding tensile properties are summarized in Table 2. All specimens exhibited the characteristic behaviour of thermoplastic polymers, consisting of an initial linear elastic region followed by yielding, attainment of ultimate tensile strength, and subsequent fracture. The slope of the initial linear region represents the tensile modulus, whereas the peak stress corresponds to the tensile strength. The elongation at break reflects the ductility of the material, and the area under the stress–strain curve is indicative of its toughness.

Virgin HDPE exhibited a tensile modulus of 1.96 GPa. In contrast, unreinforced rHDPE showed a reduced modulus of 1.62 GPa, representing a decrease of approximately 17% compared with virgin HDPE. This reduction can be attributed to polymer chain scission and structural degradation occurring during repeated processing and recycling, which adversely affect the stiffness of the material. The incorporation of nanoclay significantly improved the stiffness of rHDPE. The tensile modulus increased to 1.93 and 1.95 GPa for 0.5 and 1.0 wt% nanoclay, respectively, approaching the value of virgin HDPE. A maximum modulus of 2.39 GPa was achieved at 1.5 wt% nanoclay, corresponding to an increase of approximately 22% relative to virgin HDPE and 48% relative to neat rHDPE. This enhancement is attributed to the high intrinsic stiffness of the clay platelets and the effective restriction of polymer chain mobility through strong filler–matrix interactions. However, further increasing the nanoclay loading to 2.0 wt% reduced the modulus to 1.72 GPa, suggesting the onset of particle agglomeration and inefficient stress transfer within the matrix.

A similar trend was observed for tensile strength. Virgin HDPE exhibited a tensile strength of 30.01 MPa, whereas neat rHDPE showed a reduced strength of 25.40 MPa, corresponding to a decrease of approximately 15%. The reduction confirms the detrimental effects of recycling on the structural integrity of HDPE. The addition of nanoclay progressively improved the tensile strength, reaching 26.41 MPa at 0.5 wt% and 29.18 MPa at 1.0 wt%. The highest tensile strength of 31.27 MPa was obtained at 1.5 wt% nanoclay, exceeding that of virgin HDPE by approximately 4%. The improvement is attributed to enhanced interfacial adhesion and efficient load transfer between the polymer matrix and dispersed nanoclay platelets. Nevertheless, the tensile strength decreased to 28.01 MPa at 2.0 wt% nanoclay, indicating that excessive filler loading promotes agglomeration, which acts as stress concentration sites and initiates premature failure.

The elongation at break results reveal the influence of nanoclay on the ductility of the composites. Virgin HDPE exhibited an elongation at break of 7.9%, whereas neat rHDPE displayed a lower value of 4.3%, indicating increased brittleness after recycling. Interestingly, the composite containing 1.0 wt% nanoclay achieved the highest elongation at break (9.6%), demonstrating that an optimal filler content can simultaneously enhance strength and maintain ductility. This behaviour suggests effective dispersion of nanoclay within the polymer matrix, facilitating stress redistribution during deformation. In contrast, higher nanoclay loadings resulted in a marked reduction in elongation, decreasing to 5.1% at 1.5 wt% and 3.7% at 2.0 wt%. The reduced ductility is attributed to restricted polymer chain mobility and the formation of filler agglomerates that facilitate crack initiation and propagation. Consequently, the material undergoes a transition from ductile to brittle behaviour with increasing nanoclay content.

The tensile performance observed in this study is consistent with previous reports on nanoparticle-reinforced rHDPE systems. Sahu et al. [25] reported a 39% increase in tensile strength for rHDPE reinforced with 4 wt% carbon nanotubes, while Malyuta et al. [26] observed tensile strength improvements of up to 20.4% and stiffness enhancements of up to 93.5% in talc-filled rHDPE composites. These improvements were attributed to enhanced interfacial bonding and efficient stress transfer between the matrix and reinforcing particles. Similar mechanisms are responsible for the mechanical improvements observed in the present work. However, excessive nanoparticle addition leads to agglomeration and stress concentration effects, resulting in a decline in mechanical performance, as observed for the 2.0 wt% nanoclay composite.

Overall, the results demonstrate that nanoclay loading significantly influences the tensile behaviour of rHDPE composites. Among the investigated formulations, 1.5 wt% nanoclay provided the highest tensile strength and modulus, whereas 1.0 wt% nanoclay exhibited the greatest elongation at break and the best balance between stiffness, strength, and ductility. Therefore, the optimum nanoclay content for enhancing the tensile performance of rHDPE lies within the range of 1.0–1.5 wt%.

### 3.2. Flexural Strength

**Figure 3:**
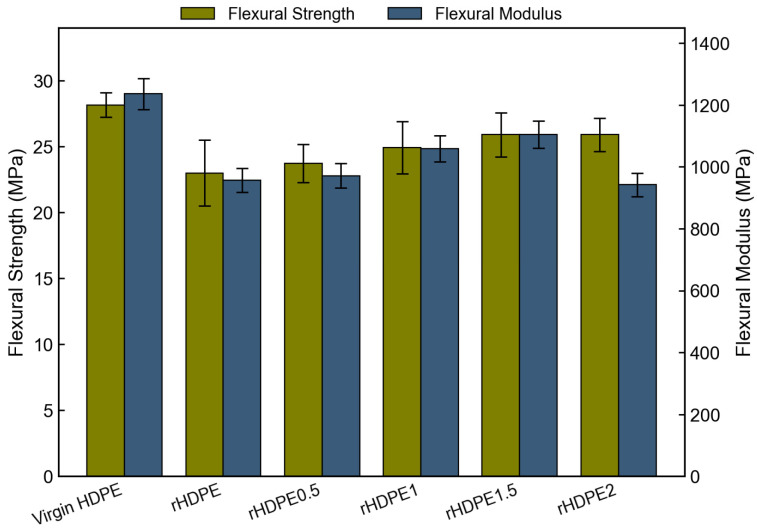
Flexural strength and modulus of rHDPE composites.

The flexural strength and flexural modulus of the rHDPE composites are presented in Figure 3 and summarised in Table 2, highlighting the influence of nanoclay reinforcement on the bending performance of recycled HDPE. Virgin HDPE exhibited a flexural strength of 28.14 MPa. In contrast, unreinforced rHDPE showed a reduced flexural strength of 22.99 MPa, corresponding to an 18.30% decrease, which can be attributed to polymer chain scission and molecular weight reduction occurring during recycling. The incorporation of nanoclay progressively enhanced the flexural strength to 23.71 MPa, 24.92 MPa, and 25.88 MPa for nanoclay loadings of 0.5, 1.0, and 1.5 wt%, respectively. No further increase was observed at 2.0 wt%, with the flexural strength remaining at 25.88 MPa. Relative to unfilled rHDPE, the maximum improvement in flexural strength was 12.57%, achieved at 1.5 wt% nanoclay.

The improvement in flexural strength can be attributed to the high aspect ratio and platelet morphology of montmorillonite nanoclay, which facilitate efficient stress transfer and hinder crack propagation under bending loads. During three-point bending, the material simultaneously experiences compressive stress on the upper surface and tensile stress on the lower surface. Well-dispersed nanoclay platelets act as reinforcing barriers across these stress fields, improving resistance to deformation and fracture. Similar improvements have been reported by Rafiee and Shahzadi [35], who observed a 15.3% increase in flexural strength in recycled polyolefin composites reinforced with organoclay. Mukaddas et al. [36] also reported enhanced flexural behaviour in nanoclay-filled polyolefin systems due to restricted polymer chain mobility and improved interfacial interactions. The plateau in flexural strength observed between 1.5 and 2.0 wt% nanoclay suggests that the reinforcing effect reached saturation, beyond which additional filler contributes little to load transfer because of increased particle agglomeration.

The flexural modulus exhibited a similar trend. Virgin HDPE showed the highest flexural modulus of 1235.75 MPa, whereas unfilled rHDPE exhibited a significantly lower modulus of 956.85 MPa, representing a reduction of 22.57%. The addition of nanoclay gradually increased the flexural modulus to 971.64 MPa at 0.5 wt%, 1059.35 MPa at 1.0 wt%, and a maximum of 1105.08 MPa at 1.5 wt%, corresponding to an improvement of 15.49% compared with neat rHDPE. This increase is attributed to the inherently high stiffness of the montmorillonite platelets and their ability to restrict polymer chain mobility when uniformly dispersed within the matrix. The trend observed in flexural modulus closely mirrors that of the tensile modulus, confirming that 1.5 wt% nanoclay provides the most effective reinforcement by promoting efficient stress transfer and restricting polymer-chain mobility, whereas excessive filler loading leads to agglomeration and deterioration of stiffness.

At a nanoclay loading of 2.0 wt%, the flexural modulus decreased to 941.84 MPa. This reduction indicates that stiffness is more sensitive to particle agglomeration than flexural strength. Excessive filler loading promotes the formation of clay clusters that interrupt the reinforcing network and create localised stress-transfer inefficiencies, thereby reducing the resistance to elastic deformation. Nevertheless, the highest modulus obtained at 1.5 wt% remained approximately 10.57% lower than that of virgin HDPE, indicating that nanoclay reinforcement can substantially recover, but not completely restore, the stiffness lost during recycling. Overall, the incorporation of nanoclay significantly improved the flexural behaviour of recycled HDPE. The composite containing 1.5 wt% nanoclay exhibited the highest flexural strength (25.88 MPa) and flexural modulus (1105.08 MPa), representing improvements of 12.57% and 15.49%, respectively, compared with neat rHDPE. The results demonstrate that moderate nanoclay loading effectively compensates for the mechanical degradation associated with recycling, while excessive loading promotes agglomeration and reduces reinforcement efficiency. Consequently, 1.5 wt% nanoclay is identified as the optimum concentration for maximising the flexural performance of rHDPE composites.

### 3.3. Impact Strength

**Figure 4:**
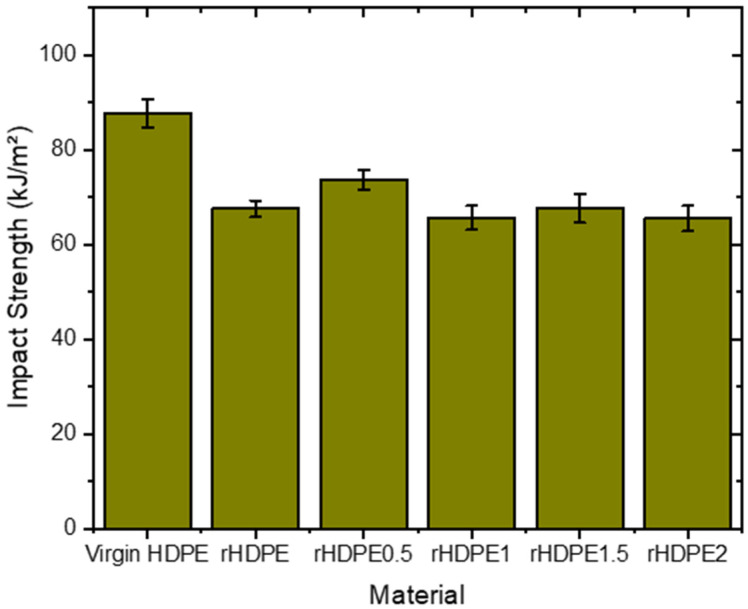
Impact strength of rHDPE composites.

The impact strength of the rHDPE composites is shown in Figure 4 and given in Table 2. The mechanical properties of the rHDPE composites exhibited distinct trends across tensile strength, flexural strength, and impact strength, indicating that the role of nanoclay depends strongly on the deformation mode. Unlike tensile and flexural loading, which benefited from improved filler-matrix interaction up to an optimum loading, impact resistance did not increase monotonically with nanoclay addition. Virgin HDPE exhibited the highest impact strength at 87.65 kJ/m^2^. Unreinforced rHDPE showed a reduction to 67.51 kJ/m^2^, a 22.98% decrease attributable to chain scission during recycling. Impact strength peaked at 0.5 wt% nanoclay loading (rHDPE0.5) at 73.58 kJ/m^2^, representing a 9.00% improvement over unreinforced rHDPE. At higher loadings, impact strength declined to 65.64 kJ/m^2^ (rHDPE1), 67.59 kJ/m^2^ (rHDPE1.5), and 65.42 kJ/m^2^ (rHDPE2). Notably, the value for rHDPE1.5 (67.59 kJ/m^2^) was statistically comparable to unreinforced rHDPE (67.51 kJ/m^2^), with the difference falling well within the respective standard deviations, and should not be interpreted as a meaningful recovery.

The observed differences stem from the distinct mechanisms underlying each property. Tensile and flexural strengths are governed by load transfer and filler-matrix interaction, which are optimised at 1.5 wt% nanoparticle loading. In contrast, impact strength depends on the material’s toughness and energy absorption capacity, which is maximised at lower nanoparticle concentrations (0.5 wt%) due to reduced voids and enhanced matrix continuity. These findings underscore the importance of tailoring nanoparticle loading based on the specific mechanical property requirements of rHDPE composites, balancing reinforcement benefits and processing challenges. These findings agree with previous studies reporting that low nanoclay loading improves toughness through better stress distribution, whereas higher loading may promote agglomeration and stress concentration, leading to reduced impact resistance [37].

### 3.4. Hardness

**Figure 5:**
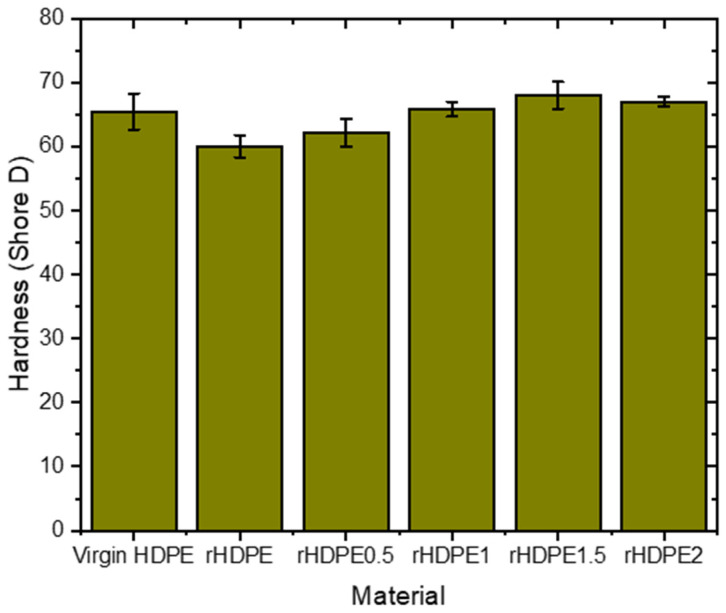
Hardness of rHDPE composites.

The hardness of rHDPE composites, shown in Figure 5 and measured on the Shore D scale, demonstrated notable improvements with nanoparticle reinforcement, following a trend similar to tensile and flexural strength but differing from impact strength. Virgin HDPE exhibited a Shore D hardness of 65.57, serving as the reference value. Recycled HDPE (rHDPE) without reinforcement showed a hardness of 60.11, representing an 8.33% reduction, likely due to the loss of polymer chain integrity and structural rigidity during the recycling process. With the addition of 0.5 wt% nanoparticles (rHDPE0.5), the hardness increased to 62.22, indicating a 3.51% improvement over unreinforced rHDPE. This enhancement can be attributed to the partial recovery of rigidity through improved matrix continuity. At 1 wt% nanoparticle loading (rHDPE1), the hardness rose to 65.92, exceeding the virgin HDPE value and representing a 9.67% improvement over rHDPE. This increase suggests that better nanoparticle dispersion enhanced the filler-matrix interaction, contributing to higher resistance to surface deformation. The highest hardness was observed at 1.5 wt% nanoparticle loading (rHDPE1.5), reaching 68.06, a 13.23% increase compared to unreinforced rHDPE and a 3.80% enhancement over virgin HDPE. This optimal performance reflects the balance between nanoparticle reinforcement and the polymer matrix’s structural integrity. However, increasing the nanoparticle concentration to 2 wt% (rHDPE2) resulted in a slight decrease in hardness to 67.08, suggesting that nanoparticle agglomeration reduced the effectiveness of the reinforcement.

Compared to tensile, flexural, and impact strength, the hardness results show a consistent improvement with increasing nanoparticle concentration up to 1.5 wt%, indicating that hardness is less sensitive to the brittleness effects caused by agglomeration at higher filler loadings. This difference arises because hardness primarily reflects surface rigidity and resistance to localised deformation rather than the energy dissipation mechanisms governing impact strength. These findings align with the trends reported by López-González et al. [38], who observed enhanced hardness in graphene-reinforced HDPE composites due to improved interfacial interactions and efficient load transfer between graphene and the polymer matrix. Their study demonstrated that careful optimization of nanoparticle dispersion could lead to significant improvements in the mechanical properties of polymer composites. Similarly, the current study highlights that nanoparticle reinforcement effectively restores and enhances the surface rigidity of rHDPE composites, particularly at optimal loading levels [39,40]. Unlike impact strength, which is sensitive to brittleness caused by agglomeration, hardness reflects surface resistance to localised deformation and is less affected by such factors.

### 3.5. Morphological Analysis

**Figure 6:**
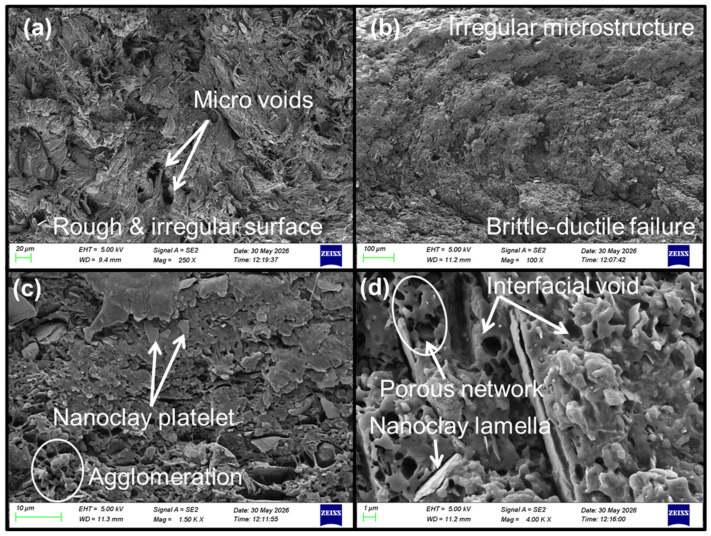
SEM micrographs of rHDPE fracture surfaces: (**a**,**b**) microvoids and granular topography in unreinforced rHDPE; (**c**) nanoclay platelet domains in rHDPE1.5; (**d**) nanoclay lamellae and interfacial voids in rHDPE2.

Scanning electron microscopy was conducted on fractured surfaces of the rHDPE composites and the results are presented in Figure 6. Figure 6a shows the fracture surface of unreinforced rHDPE, which presents a relatively rough and granular topography with a densely packed, irregular microstructure, consistent with the brittle-to-ductile mixed fracture behaviour typical of thermally degraded recycled polyolefins. Figure 6b reveals a layered, foliated texture with visible microvoids and inter-laminar discontinuities, which are characteristic of chain scission and partial delamination arising from thermo-oxidative degradation during reprocessing. These features are consistent with the reduction in tensile and impact strength observed in unreinforced rHDPE relative to virgin material.

Figure 6c shows that the nanoclay-reinforced composite surface exhibits a more fragmented, platelet-like morphology with flake-shaped domains distributed across the matrix. This texture is indicative of partially exfoliated or intercalated nanoclay layers within the rHDPE matrix. The presence of such morphology at lower nanoclay loadings (≤1.5 wt%) supports the improved tensile and flexural strength observed in these formulations, as partially exfoliated platelets provide an extended filler–matrix interfacial area that promotes effective stress transfer. However, some localised clustering of platelet fragments is also visible, suggesting the onset of agglomeration at higher filler concentrations, consistent with the mechanical performance plateau observed at 2 wt% loading.

At the highest magnification (Figure 6d), the fracture surface of the reinforced composite reveals a highly porous, sponge-like morphology interspersed with elongated nanoclay platelets and irregular cavity formations. The porous network is likely attributable to void formation at poorly bonded filler–matrix interfaces and to gas entrapment during melt processing. The elongated platelet-shaped features visible at this scale confirm the presence of nanoclay lamellae within the polymer matrix and provide direct morphological evidence of filler incorporation. The coexistence of porous regions and embedded platelets suggests that while nanoclay reinforcement was achieved, complete exfoliation was not attained, and interfacial debonding contributed to the energy dissipation mechanism observed in the impact strength results. Overall, the SEM observations corroborate the mechanical data, supporting the conclusion that 1.5 wt% nanoclay loading provides the most favourable balance between filler dispersion and matrix integrity in the rHDPE system investigated.

### 3.6. Life Cycle Assessment (LCA)

**Figure 7:**
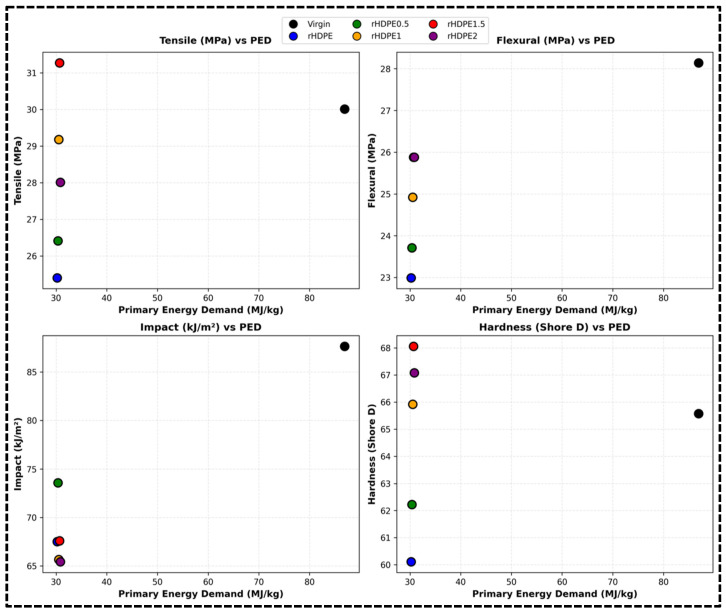
Mechanical Properties vs. PED (Tensile, Flexural, Impact, Hardness).

The environmental performance of the HDPE-nanoclay composites was first assessed using primary energy demand (PED), as illustrated in Figure 7. A clear distinction is observed between virgin HDPE and all recycled HDPE (rHDPE) formulations. Virgin HDPE exhibits the highest PED value of 86.92 MJ/kg (=82.0 MJ/kg material PED × 1.06 processing multiplier; cf. Table 1), consistent with the energy-intensive processes involved in steam cracking, monomer production, and polymerisation. In contrast, the rHDPE-based formulations fall within a narrow range of 30.21–30.84 MJ/kg, confirming the substantial energy savings achieved through mechanical recycling. The slight increase in PED with increasing nanoclay loading reflects the higher embodied energy of the clay phase, but the effect remains small because the filler content is limited to 2 wt%. Figure 6 further shows that substantial improvements in tensile strength, flexural strength, and hardness were achieved without a meaningful energy penalty, especially for rHDPE1.5.

**Figure 8:**
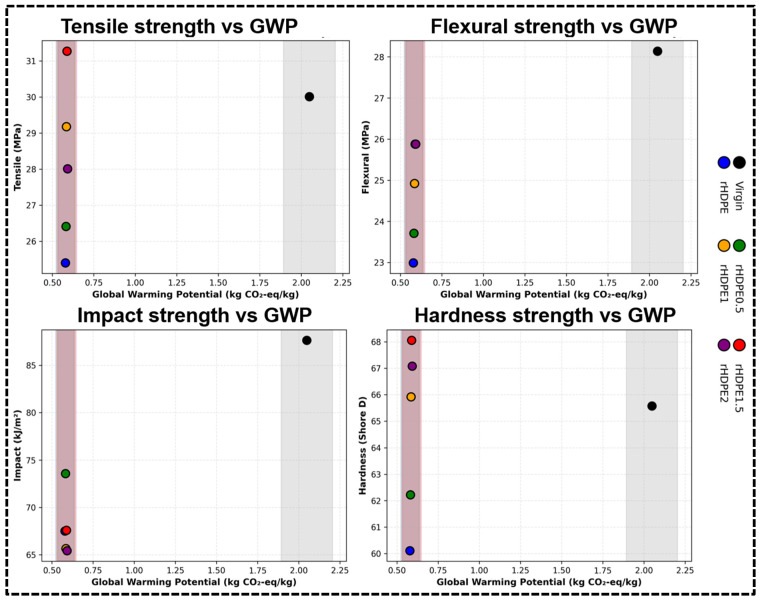
Mechanical properties plotted against GWP with source-based uncertainty bands (±7.7% for virgin HDPE, ±10% for recycled HDPE and nanoclay, and ±6% for processing electricity).

To deepen the environmental analysis, Figure 8 presents the global warming potential (GWP) of the composites together with uncertainty bands derived from the source ranges used in the LCA model. Virgin HDPE shows the highest GWP at 2.0484 kg CO_2_-eq/kg (=1.95 kg CO_2_-eq/kg material GWP + 0.0984 kg CO_2_-eq/kg processing contribution; cf. Table 1), whereas the recycled formulations remain clustered within a much lower range of 0.5784–0.5918 kg CO_2_-eq/kg. This large reduction confirms that the dominant environmental benefit arises from replacing virgin HDPE with recycled material. The gradual increase in GWP from rHDPE to rHDPE2 is attributable to the increasing nanoclay fraction and its own embodied burden; however, this increment is modest because the filler loading is low. The tight clustering of the recycled formulations indicates that the environmental ranking among the recycled grades is driven mainly by mechanical performance rather than by large differences in embodied carbon.

**Figure 9:**
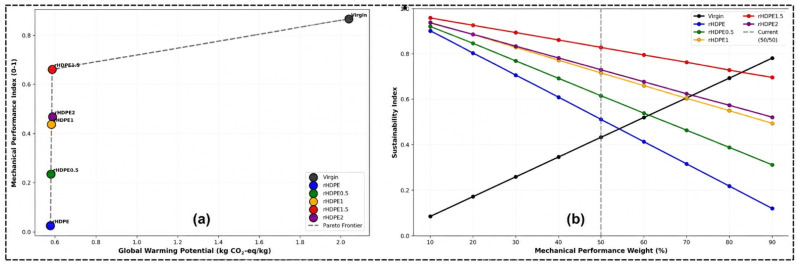
(**a**)—Pareto Frontier (GWP vs. Mechanical Index) and (**b**)—Weighting Sensitivity (SI vs. Mechanical Weight).

To better understand the performance trade-offs, Figure 9a integrates mechanical performance into a Pareto-front analysis. The results reveal that rHDPE1.5 lies closest to the desirable region of high mechanical performance and low environmental impact. Virgin HDPE, although mechanically strong, is displaced by its substantially higher carbon footprint and PED. Figure 9b further evaluates whether the sustainability ranking is sensitive to changes in stakeholder priorities. The equal weighting (0.5:0.5) adopted in the Sustainability Index (SI) was selected as a neutral baseline in which mechanical performance and environmental performance were considered equally important. Because the relative importance assigned to these criteria may vary among stakeholders and applications, a weighting sensitivity analysis was conducted across a broad range of mechanical weightings (10–90%). Across most of the weighting range examined (10–80% mechanical weighting), rHDPE1.5 remained the highest-ranked option, indicating that its favourable balance between performance and environmental burden was robust and not an artefact of the selected baseline weighting. Only at the extreme condition of 90% mechanical weighting did virgin HDPE rank first, as its slightly higher mechanical index outweighed its environmental penalty under a scenario in which environmental considerations were assigned minimal importance. These results demonstrate that rHDPE1.5 remains the most eco-efficient formulation under balanced and environmentally informed decision-making scenarios, while also confirming the robustness of the sustainability ranking to variations in weighting assumptions.

**Figure 10:**
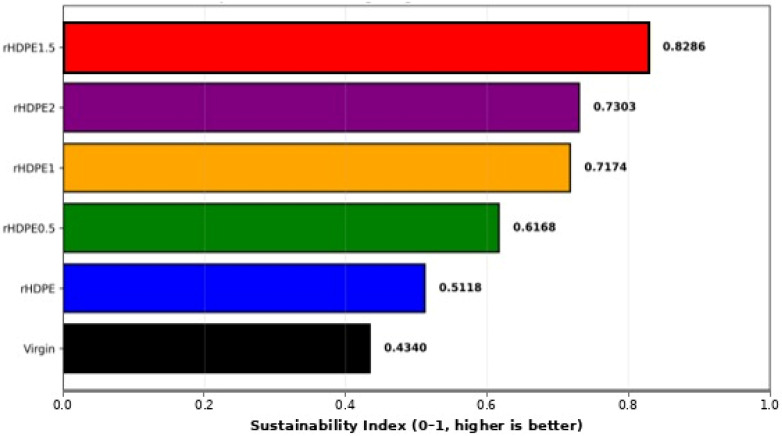
Sustainability Index ranking of all materials.

The final sustainability assessment, shown in Figure 10, combines mechanical and environmental indicators into a single Sustainability Index (SI). Virgin HDPE achieves the lowest SI (0.4340) because its strong mechanical attributes are outweighed by its high PED and GWP burdens. All rHDPE-based composites achieve higher SI values, ranging from 0.5118 to 0.8286. Notably, rHDPE1.5 exhibits the highest SI (0.8286), identifying it as the most balanced and sustainable formulation. This reflects the synergistic effect of nanoclay at this loading, where tensile strength, hardness, and flexural rigidity improve significantly, while the environmental penalty remains very small. rHDPE2 and rHDPE1 rank second and third, respectively, supporting the conclusion that the optimum nanoclay range lies around 1–2 wt%.

The stability of these conclusions is reinforced by the sensitivity analyses. First, a ±20% variation in the environmental inputs did not alter the relative ranking of the materials, with rHDPE1.5 remaining the top-performing formulation in all scenarios examined. Second, the weighting sensitivity analysis showed that rHDPE1.5 remained the most sustainable formulation across most of the tested mechanical-environmental priority ranges, but at 90% mechanical weighting, virgin HDPE ranked first. Together, these results indicate that the sustainability ranking is robust within the assumptions and variation bounds used in the present analysis, although the final ranking can shift under an extreme mechanically dominated weighting scenario.

Although the present study primarily focused on mechanical and sustainability performance, additional polymer-specific characterization such as DSC, XRD, and GPC would provide a deeper understanding of crystallinity evolution, molecular weight variation, and structure–property relationships in recycled HDPE nanocomposites. These analyses are recommended for future investigation.

## 4. Conclusions

This study demonstrates the effectiveness of nanoclay reinforcement in improving the mechanical performance and sustainability of recycled high-density polyethylene (rHDPE) composites. The incorporation of nanoclay successfully compensated for the degradation in properties caused by recycling, resulting in significant enhancements in strength, stiffness, and hardness. Among the formulations investigated, the composite containing 1.5 wt% nanoclay exhibited the optimum performance, achieving increases of 23.11% in tensile strength, 12.57% in flexural strength, and 13.23% in hardness relative to unfilled rHDPE. Furthermore, the tensile and flexural moduli reached their highest values of 2.39 GPa and 1105.08 MPa, representing improvements of 47.53% and 15.49%, respectively, compared with neat rHDPE. The environmental assessment confirmed the sustainability benefits of recycling. The cradle-to-gate life cycle assessment revealed that virgin HDPE exhibited the highest environmental impacts, with a global warming potential of 2.0484 kg CO_2_-eq/kg and cumulative energy demand of 86.92 MJ/kg. In contrast, all recycled formulations showed substantially lower impacts, ranging from 0.5784 to 0.5918 kg CO_2_-eq/kg and 30.21–30.84 MJ/kg, respectively. Integrating the mechanical and environmental indicators through a Sustainability Index identified the 1.5 wt% nanoclay composite as the most balanced formulation, achieving the highest index value of 0.8286. Overall, the results demonstrate that the strategic incorporation of low nanoclay loadings can not only restore but also surpass the mechanical performance of virgin HDPE while maintaining the significant environmental advantages associated with polymer recycling. The developed rHDPE/nanoclay composites therefore represent a promising sustainable material for applications in packaging, construction, and automotive components, supporting circular economy objectives and resource-efficient material development.

## Figures and Tables

**Table 1:** Environmental Inventory Data for LCA Calculations.

Material	GWP (kg CO_2_-eq/kg)	GWP Range	PED (MJ/kg)	PED Range	Data Source
Virgin HDPE	1.95	1.80–2.10	82.0	78–86	[28,29]
Recycled HDPE	0.48	0.43–0.53	28.5	25.7–31.3	[28,30]
Nanoclay	1.15	1.04–1.26	58.0	52.2–63.8	[31,32]

**Table 2:** Tensile properties of virgin HDPE, rHDPE, and rHDPE/nanoclay composites.

Material (Nanoclay wt%)	Tensile Strength (MPa)	Tensile Modulus (GPa)	Elongation at Break (%)	Flexural Strength (MPa)	Flexural Modulus (MPa)	Impact Strength (kJ/m^2^)	Hardness (Shore D)
Virgin HDPE	30.01 ± 1.74	1.96 ± 0.12	7.9 ± 0.63	28.14 ± 0.93	1235.75 ± 63.25	87.65 ± 2.94	65.57 ± 2.83
rHDPE (0%)	25.40 ± 1.81	1.62 ± 0.96	4.3 ± 0.42	22.99 ± 2.49	956.85 ± 54.74	67.51 ± 1.72	60.11 ± 1.74
rHDPE + 0.5%	26.41 ± 2.30	1.93 ± 0.15	5.2 ± 0.47	23.71 ± 1.46	971.64 ± 58.45	73.58 ± 2.10	62.22 ± 2.18
rHDPE + 1.0%	29.18 ± 2.47	1.95 ± 0.18	9.6 ± 0.75	24.92 ± 1.98	1059.35 ± 61.12	65.64 ± 2.58	65.92 ± 1.14
rHDPE + 1.5%	31.27 ± 2.13	2.39 ± 0.58	5.1 ± 0.58	25.88 ± 1.66	1105.08 ± 66.34	67.59 ± 3.02	68.06 ± 2.11
rHDPE + 2.0%	28.01 ± 3.16	1.72 ± 0.36	3.7 ± 0.36	25.88 ± 1.27	941.84 ± 52.45	65.42 ± 2.70	67.08 ± 0.63

## Data Availability

Data will be made available on request.
